# Extraction, Chemical Composition, and Antifungal Activity of Essential Oil of Bitter Almond

**DOI:** 10.3390/ijms17091421

**Published:** 2016-08-29

**Authors:** Huiling Geng, Xinchi Yu, Ailin Lu, Haoqiang Cao, Bohang Zhou, Le Zhou, Zhong Zhao

**Affiliations:** 1 College of Science, Northwest A & F University, Yangling 712100, Shaanxi, China; yuxinchi121@163.com (X.Y.); ailin_lu@163.com (A.L.); 18700809193@163.com (H.C.); zhoubohang@nwsuaf.edu.cn (B.Z.); 2 Key Laboratory of Environment and Ecology in Western China of Ministry of Education, Yangling 712100, Shaanxi, China

**Keywords:** bitter almond, essential oil, GC-MS, chemical composition, antifungal activity

## Abstract

The essential oil from the powder residual of dried bitter almond, a novel and environmentally-friendly fungicide, was successfully extracted in a 0.7% yield by hydro-distillation under optimized conditions. The chemical composition of bitter almond essential oil (BAEO) was analyzed by gas chromatography–mass spectrometry (GC–MS). Twenty-one different components representing 99.90% of the total essential oil were identified, of which benzaldehyde (62.52%), benzoic acid (14.80%), and hexadecane (3.97%) were the most abundant components. Furthermore, the in vitro and in vivo antifungal activities of BAEO against common plant pathogenic fungi were evaluated by the mycelium linear growth rate method and pot test, respectively. It was documented that 1 mg/mL of BAEO could variously inhibit all tested pathogenic fungi with the inhibition rates of 44.8%~100%. Among the tested 19 strains of fungi, the median effective concentration (EC_50_) values of BAEO against *Alternaria brassicae* and *Alternaria solani* were only 50.2 and 103.2 μg/mL, respectively, which were higher than those of other fungi. The in vivo antifungal activity of BAEO against *Gloeosporium orbiculare* was much higher than *Blumeria graminis*. The protective efficacy for the former was up to 98.07% at 10 mg/mL and the treatment efficacy was 93.41% at 12 mg/mL. The above results indicated that BAEO has the great potential to be developed as a botanical and agricultural fungicide.

## 1. Introduction

Plant pathogenic fungi have always been recognized as one of the main casual agents of plant diseases, which could infect any tissue at different stages of plant growth as well as result in a frustrating decline in quality and quantity of agricultural products. An investigation presented by Agrios’ group discloses that, in 2000, pathogenic fungi alone caused a nearly 20% reduction in the yield of major food and cash crops [1,2]. Furthermore, plant mycosis is notorious for its potential to cause substantial yield losses for global agriculture and its strongly detrimental effect on the food industry. Furthermore, many phytopathogenic fungi represent a serious risk for animal and human health because of the production of mycotoxins, a class of harmful secondary metabolites [3]. In this context, a series of chemical fungicides with different frameworks have been developed with the aim to address this intractable problem. Although the commercial fungicides are highly effective to control plant diseases, a series of health and environmental problems are associated with continuing application of synthetic fungicides, such as high toxicity, drug resistance, residue persistence, and so on [4]. Based on these problems caused by chemical fungicides, in the past decades, researchers have switched their attention to natural product-based or -derived antimicrobial agents due to their lower environmental and mammalian toxicity.

As one of the most promising groups of natural products, essential oils are made up of many different volatile compounds and considered as potential targets for the development of safe natural antifungals [5,6]. In recent years, more and more plant essential oils have been documented to have the ability to effectively inhibit the growth of phytopathogenic fungi in vitro and in vivo [7,8,9,10,11]. Given that we have explored the extraction, chemical composition and biological activity of fatty oil from bitter almond [12,13,14,15,16,17]; here, we extracted and analyzed the composition of bitter almond essential oil (BAEO), and further investigated its potential as antifungal agent.

*Armeniaca* *sibirica* (L.) Lam, commonly named as wild apricot, belongs to the *Armeniaca* genus of the Rosaceae family. It is a perennial deciduous tree and mainly cultivated in Asia, Europe, and America [18,19]. Bitter almond is the dried mature seed of *Prunus armeniaca* L., *Armeniaca sibirica* (L.) Lam var. *sibirica*, and *Prunus* *mandshurica* (Maxim.) Koehne, and related species. As a traditional oriental medicine, apricot seed exhibits a wide range of biological activities including antihyperlipidemia, anti-inflammatory, anticancer, antioxidant, antimicrobial, antiasthmatic, analgesic, preventing heart disease, atherosclerotic, etc. [20,21,22,23,24]. Consequently, it has been widely used to cure respiratory disorders (coughing, wheeze, asthma, emphysema, and bronchitis) and skin diseases (acne vulgaris, dandruff, and furuncle) [25]. Bitter almond is rich in a variety of nutrients, such as oils, proteins, vitamins, amino acids, etc. [26]. The oil of bitter almond is classified as a fatty oil and an essential oil, the former consists mainly of unsaturated aliphatic acids while the latter is made up of volatile compounds. After pressing the fatty oil from the dried powder of bitter almond, the residual (which contains the essential oil) is often discarded as a waste. In order to make full use of the residual and to alleviate environmental pollution, the essential oil has been extracted from the powder residual in a 0.7% yield. Although BAEO has been extensively used as an additive or nutritious ingredient in food, beverages, health care products, cosmetics, and pharmaceuticals [19,27], little is known about its chemical composition and antifungal activity.

With the aim of searching for new antifungal agents and maximizing the use of natural resources, the present work focuses on the study of the essential oil from bitter almond: its extraction, chemical composition, and in vitro and in vivo antifungal activity against common plant pathogenic fungi.

## 2. Results and Discussion

### 2.1. Extraction of BAEO

**Figure 1:**
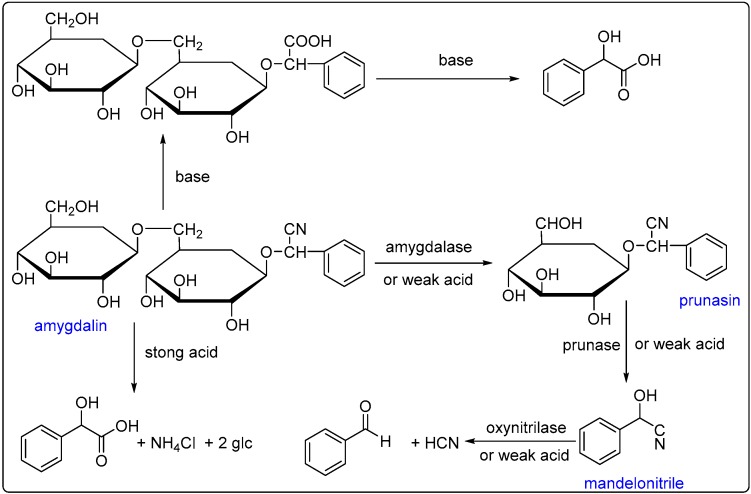
Hydrolysis of amygdalin.

After removing the fatty oil from the dried powder of bitter almond via the heating-crushing method, BAEO was extracted in a 0.7% yield from the left press cake by the hydro-distillation method using a special apparatus designed by our group. The optimized extracting process was accomplished as follows: firstly, the powder residual (100 mg) was soaked in 500 mL of weak acid water (pH = 5) at 40 °C for 1 h, so that the main bioactive substance, amygdalin, could be totally hydrolyzed to give glucose and benzaldehyde meanwhile releasing the poisonous constituent, hydrogen cyanide (HCN) (Figure 1). The resulting suspension was then vigorously stirred and distilled for 1 h, and the yellowish BAEO accumulated at the bottom of the handmade apparatus, which was designed to extract volatile oil. After the water–oil mixture had been standing in a separatory funnel for 30 min, crude BAEO was separated from the lower organic layer, and dried with anhydrous sodium sulfate. Finally, BAEO was achieved in a 0.7% yield.

### 2.2. Chemical Composition of BAEO

The chemical composition of the BAEO was evaluated by gas chromatography–mass spectrometry (GC–MS) analysis (Table 1). Among the confirmed 21 types of compounds accounting for 99.90% of the total essential oil, the main chemical constituents were identified as benzaldehyde (62.52%), benzoic acid (14.80%), and hexadecane (3.97%). The other 18 components, eicosane (2.26%), mesitylene (1.62%), 1-ethyl-3-methyl-benzene (1.61%), benzyl alcohol (1.59%), 1,3-xylene (1.56%), dodecane (1.50%), 3,5-di-*tert*-butylphenol (1.24%), 3-(hydroxyl-phenyl-methyl)-2,3-dimethyl-octan-4-one (1.23%), dodecyl aldehyde (1.18%), benzyl cyanide (1.06%), 1,4-xylene (0.90%), ethylbenzene (0.69%), tetradecane (0.61%), 3-ethyl-2-undecanone (0.50%), heptadecane (0.39%), 2-methylundecane (0.26%), 1,2,4-trimethylbenzene (0.24%), and 1,2-xylene (0.18%), were identified in lesser amounts. Furthermore, a few unknown compounds were found as trace or minor components.

Although the chemical composition of the essential oil of apricot seed has only ever been analyzed by Lee and colleagues using GC–MS as well, only the most abundant compounds, benzaldehyde (90.6%), mandelonitrile (5.2%) and benzoic acid (4.1%), were identified [28]. The different results mentioned above may be attributed to the diversity of bitter almond birthplaces and different isolation conditions used in our laboratory. The apricot fruit in their test was picked from Gyeongju in Korea, however, in our test, it was harvested from Linyou county, Shaanxi province in China. We speculate that the differences in the climates of Korea and China may result in the high content of benzaldehyde in their test. Moreover, the reason that we did not detect any mandelonitrile was that it had been thoroughly decomposed to benzaldehyde and HCN under weak acid condition (see Figure 1).

### 2.3. The in Vitro Antifungal Activity

To the best of our knowledge, up to now, only a couple of research groups have ever tentatively explored the antibacterial activity of the essential oil of apricot seed [28] and the antifungal activity of BAEO aqueous solution [29]. There is no research involving the systematic investigation of the potential antifungal activity of BAEO, therefore, we aimed to evaluate the in vitro antimicrobial activity of BAEO against 19 strains of common phytopathogenic fungi by the mycelium linear growth rate method and in vivo antifungal activity against *Gloeosporium orbiculare* (Berk.) Berk and *Blumeria graminis* (DC.) E.O. Speer f. sp. *tritici* Em. Marchal via pot test.

The in vitro antifungal activities of BAEO, benzaldehyde, and kresoxim-methyl (a commercially available fungicide), were screened against 19 strains of plant pathogenic fungi at the concentration of 1 mg/mL using the above mentioned method previously reported by our group [30]. Comparison of the antifungal activities of these three agents (see Table 2) shows that BAEO not only exhibits medium to high antifungal activity in all cases, but also presents a broad-spectrum antifungal activity. In contrast, the inhibition rates of BAEO ranged from 44.8% to 100%, while those of benzaldehyde and kresoxim-methyl were 28.6%~100% and 63.0%~100%, respectively. The inhibition rates of BAEO against 15 out of 19 fungi were higher than 50%. In particular, for *Alternaria solani*, *Gloeosporium* *orbiculare*, *Gaeumannomyces graminis*, *Botrytis* *cinerea*, and *Alternaria brassicae*, the inhibition rates of BAEO were unexpectedly up to 100%. Compared with the activities of benzaldehyde, the inhibition rates of BAEO against 14 out of 19 fungi were greater. It is worth noting that the activities of BAEO against *Fusarium oxysporum* sp. *cucumebrium*, *Pyricular Oryzae cavgra*, *Botrytis* *cinerea*, and *Fusarium oxysporum* f. sp. *vasinfectum* even surpassed those of kresoxim-methyl. In most cases, there were significant differences between the activities of the three tested compounds (*p* < 0.05).

In order to further explore the antifungal potential of BAEO, the essential oil and kresoxim-methyl were both subjected to an antifungal toxicity assay to determine their median effective concentration (EC_50_) against 19 strains of fungi. The corresponding results are shown in Table 3 and Table 4, respectively.

It can be clearly seen from Table 3 that BAEO displayed antimicrobial activity in varying degrees against the tested fungi. The EC_50_ values of BAEO fluctuated between 50.2 and 642.0 μg/mL. BAEO revealed the highest activity against *Alternaria brassicae* with an EC_50_ value of 50.2 μg/mL; on the contrary, the lowest activity was observed for *Alternaria alternata* (EC_50_ = 642.0 μg/mL). The EC_50_ values of BAEO against seven out of 19 fungi were lower than 300 μg/mL, while EC_50_ values against 10 out of 19 fungi were higher than 500 μg/mL. The top three highest activity levels were observed for *Alternaria brassica*, *Alternaria* *solani*, and *Gaeumannomyces graminis*, and the corresponding EC_50_ values were 50.2, 103.2, and 192.0 μg/mL, respectively. The bottom three lowest activity levels were found for *Alternaria alternata* (EC_50_ = 642.0 μg/mL), *Fusarium* *graminearum* (EC_50_ = 627.5 μg/mL), and *Valsa mali* (EC_50_ = 610.8 μg/mL).

### 2.4. Effect of Treatment Time on the In Vitro Antifungal Activity of BAEO

To explore the effect of treatment time on the activity of BAEO, the antifungal activities of BAEO and kresoxim-methyl against *Fusarium oxysporum* f. sp. *niveum* and *Alternaria* *solani* were tested at the concentrations of 0.5 and 1.0 mg/mL by the mycelium linear growth rate method. Antifungal activities were measured at 24 h, 48 h, and 72 h post-treatment, respectively, and recorded in Table 5.

**Figure 2:**
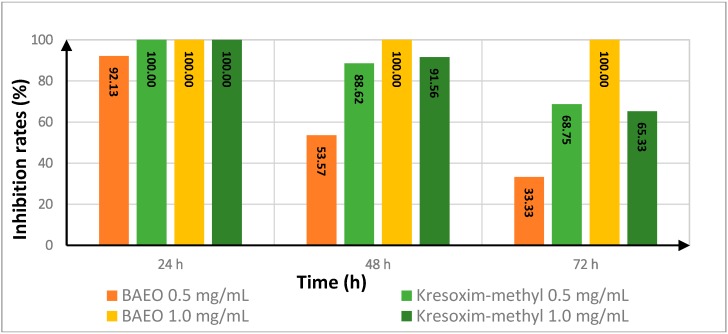
Effect of treatment time on the antifungal activity of BAEO against *Fusarium oxysporum* f. sp. *niveum*.

**Figure 3:**
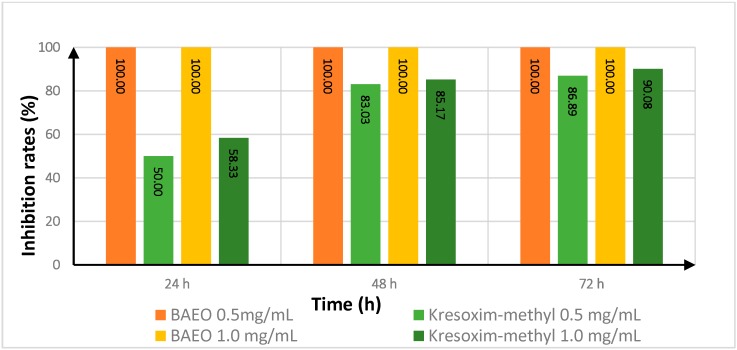
Effect of treatment time on the antifungal activity of BAEO against *Alternaria* *solani*.

Generally speaking, except for the results of 0.5 mg/mL of BAEO against *Fusarium oxysporum* f. sp. *niveum*, the inhibition rates of BAEO against two tested fungi did not decrease at all and always maintained 100% inhibition rates even at 72 h post-treatment (Table 5, Figure 2 and Figure 3). Although, at 0.5 mg/mL, the activities of BAEO against *Fusarium oxysporum* f. sp. *niveum* were a little lower than those of kresoxim-methyl, the activities of BAEO at 1.0 mg/mL were completely opposite. Compared with the activities for the tested fungi, the activities for *Fusarium oxysporum* f. sp. *niveum* gradually reduced with the prolongation of time, whereas the activities for *Alternaria* *solani* dramatically increased under the same circumstances. Particularly, whether for *Fusarium oxysporum* f. sp. *niveum* or for *Alternaria* *solani*, the efficacy of BAEO at 1.0 mg/mL was much better than kresoxim-methyl—even 72 h later, it was still up to 100%. Compared with the antifungal activity of the standard drug against *Alternaria* *solani*, the antifungal activity of BAEO was sustained at 100% from the beginning of the trail to the end. It was confirmed that, in most cases, the efficacy of BAEO was much higher than kresoxim-methyl. Indeed, the extension of treatment time from 24 h to 72 h did not affect the efficacy of BAEO, whereas the antifungal activity of kresoxim-methyl quickly decreased.

### 2.5. In Vivo Antifungal Activity

*Gloeosporium orbiculare* and *Blumeria graminis*, the ubiquitous plant mycosis, often lead to lethal damage to cucumber and wheat. For the purpose of evaluating the control effect of BAEO against the two strains of fungi, the in vivo antifungal activity evaluations of BAEO were carried out by pot test, in order to confirm whether it was appropriate to protect the growth of plants from antifungal infection. 

**Figure 4:**
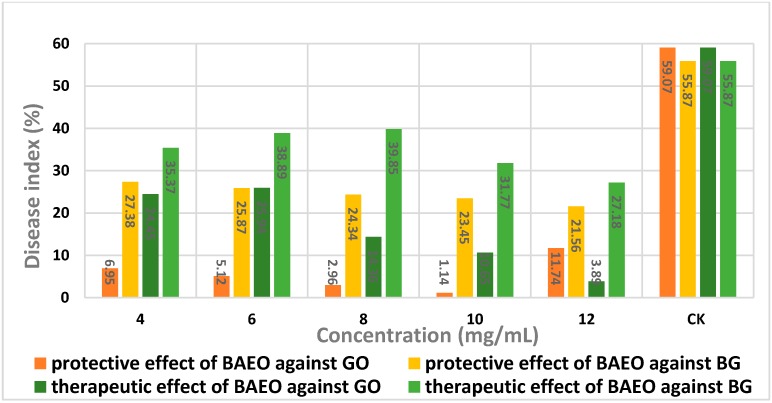
Disease index of cucumber and wheat seedlings. GO, *Gloeosporium orbiculare*; BG, *Blumeria graminis.*

**Figure 5:**
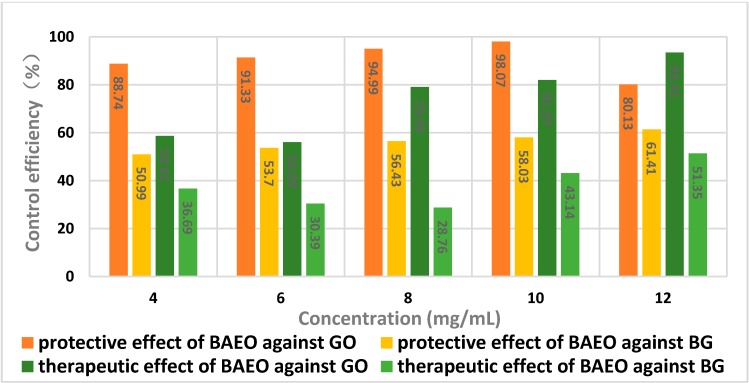
Protective and therapeutic efficacy of BAEO against *Gloeosporium orbiculare* (GO) and *Blumeria graminis* (BG).

By comparing the protective effect of BAEO (Table 6) against *Gloeosporium orbiculare* with that of therapeutic effect (Table 7), in general, a tendency of gradually increasing activity was observed in most cases when the concentration of BAEO was increased from 4 to 12 mg/mL (Figure 4 and Figure 5); the growth trend of the former was much faster than that of the latter, hence BAEO could be utilized to protect cucumber from fungal invasion. There was an abnormal phenomenon for *Gloeosporium* *orbiculare* in that the protective efficacy of BAEO at 12 mg/mL was less than that of BAEO at 10 mg/mL, which presumably indicates that higher concentrations of BAEO may lead to tissue damage and infection spread. Based on these findings, it is likely that the most effective concentration of BAEO is 10 mg/mL, making the leaf blades of cucumber resist the fungal infection.

As far as *Blumeria graminis* was concerned (Table 8 and Table 9, Figure 4 and Figure 5), the protective efficacy of BAEO was slightly better than its therapeutic efficacy at all tested concentrations. Similarly, in considering the situation of BAEO being employed against *Gloeosporium orbiculare*, the protective efficacy and the therapeutic efficacy were both positively correlated with the concentration of BAEO.

The results from Table 6, Table 7, Table 8 and Table 9 and Figure 4 and Figure 5 suggested that the control efficacy of BAEO for *Gloeosporium orbiculare* was better than that of *Blumeria graminis*. Furthermore, the protective action of BAEO for *Gloeosporium orbiculare* was much stronger than that of *Blumeria graminis*, while the therapeutic action for the former was a little higher than that of the latter. By carefully observing the phenomena of the blades after spraying BAEO, we found that it immediately formed an oil membrane layer on the leaf blade surface in the protection group. We speculated that the oil membrane layer could effectively prevent the pathogen spores from penetrating into the internal tissue, thereby reducing the incidence of infection.

## 3. Materials and Methods

### 3.1. General

Unless otherwise mentioned, all reagents and solvents were purchased from commercial suppliers and used without further purification, and were of analytical reagent grade. Kresoxim-methyl, a commercial fungicide, benzaldehyde standard, and dimethyl sulfoxide (DMSO) were purchased from J&K Chemical Ltd. (Beijing, China). The water used was redistilled and ion-free.

### 3.2. Materials

**Bitter almond.** Wild apricot fruits (*Armeniaca* *sibirica* (L.) Lam) were harvested from Linyou county, Shaanxi province, China. Later, bitter almonds were dried in the shade.

**Fungi.** Nineteen strains of tested plant pathogenic fungi, *Fusarium oxysporum* sp. *cucumebrium* Owen (FOC), *Valsa mali* Miyabe et Yamade (VM), *Pyricularia oryzae cavgra* (PO), *Fusarium graminearum* (FG), *Alternaria alternata* (Fr) Keissler (AA), *Alternaria solani* (AS), *Phytophthora capsici* Leonian (PC), *Gloeosporium fructigenum* (GF), *Fusarium oxysporum* f. sp. *lycopersici* Synder et Hansen (FOL), *Gloeosporium orbiculare* (GO), *Verticillium dahliae* Kleb (VD), *Gaeumannomyces graminis* var. *tritici* (GG), *Botrytis* *cinerea* (BC), *Fusarium oxysporum* f. sp. *vasinfectum* (FOV), *Curvularia lunata* (CL), *Fusarium* *oxysporum* *(*Schlecht.) (FOS), *Colletotrichum gloeosporioides* (Penz.) et Sacc. (CG), *Fusarium oxysporum* f. sp. *niveum* (FON), *Blumeria gramini*s f. sp. *tritici* (BG), and *Alternaria brassicae* (AB), were provided by the Center of Pesticide Research, Northwest A&F University, China. These fungi were grown on potato dextrose agar (PDA) plates at 28 °C and maintained at 4 °C with periodic subculturing.

### 3.3. Extraction of BAEO

Dried bitter almonds were smashed to powder using a small grinder. After isolating the fatty oil from the powder by the heat-crushing method [16], the essential oil was acquired from the residual by hydro-distillation using a special apparatus designed by our group. It was dried over anhydrous Na_2_SO_4_, filtered, kept in amber glass bottle, and stored at 4 °C before testing.

### 3.4. Analysis of the Chemical Composition of BAEO

The chemical composition of BAEO was identified by a Shimadzu QP2010 Ultra GC–MS (Shimadzu Co., Kyoto, Japan). The essential oil (10 μL) was dissolved in dichloromethane (1000 μL) and 3 μL of the diluted solution was injected using a splitless mode. The capillary column was a DB-WAX model with the length of 30 m, a diameter of 0.25 mm, and a film thickness of 0.25 μm (Agilent Technologies, Santa Clara, CA, USA). Helium was used as the carrier gas at a flow rate of 1 mL/min. The column inlet pressure was 57.3 kPa. The oven temperature was maintained at 240 °C. The column temperature was programmed as follows: initial temperature of 60 °C for 4 min, gradually increased to 100 °C at 5 °C/min, then warmed up to 240 °C at 10 °C/min and held for 5 min. Injector and detector temperatures were kept at 230 °C. Electronic ion (EI) mode was set as 77.3 eV, while mass spectra were recorded in the 50–500 amu range and the ion source-temperature was 200 °C. The chemical components of BAEO were quantified by relative percent of peak area from the MS signal and identified by comparing their mass spectra with those stored in the spectrometer database using NIST14.LIB (Shimadzu Co., Kyoto, Japan).

### 3.5. Assay of the in Vitro Antifungal Activity of BAEO

In vitro antifungal activity was assayed by the mycelium linear growth rate method previously reported [30]. All tested fungi maintained on PDA medium slants were subcultured for 48 h in Petri dishes prior to testing and used for inoculation of fungal strains on PDA plates. The tested compound (0.4 mL of BAEO or benzaldehyde) was completely dissolved in 3.6 mL of 5% DMSO to prepare the stock solution. Then 1 mL of the resulting solution was added to 99 mL of melted PDA agar at 50 °C, quickly and completely mixed, and poured into a Petri dish in a laminar flow chamber. The final concentration of the test compounds in the culture medium was 1 mg/mL for the activity screening test, and the final concentration of DMSO was 0.05%, which was proven to have no significant effect on fungal growth. Kresoxim-methyl (1 mg/mL) in the culture medium containing 0.5% DMSO (*v*/*v*) and 0.5% DMSO in the culture medium were used as a positive control and a blank control, respectively. When the medium in the plate was partially solidified, a 5-mm thick and 4-mm diameter disc of fungus cut from previously subcultured Petri dishes were placed at the center of the semisolid medium. The dishes were kept in an incubator at 28 °C for 72 h. Each experiment was carried out in triplicate. The diameters (in mm) of inhibition zones were measured with a caliper in three different directions and the growth inhibition rates were calculated according to the following formula and expressed as means ± standard deviation (SD).

Inhibition rate (%) = [(*d_c_* − *d*_0_) − (*d_s_* − *d*_0_)]/(*d_c_* − *d*_0_) × 100 (1)

where *d_0_* is the diameter of the fungus cut, *d_c_* represents the diameter of a fungal colony in the blank test, and *d_s_* refers to the diameter of a fungal colony in the compound-treated test.

According to the results of in vitro antifungal activity, BAEO and kresoxim-methyl were further subjected to determine their EC_50_ by the same method. A stock solution of them was prepared in 5% DMSO aqueous solution, and then diluted by 5% DMSO in water using the serial two-fold dilution method to obtain a series of stock solutions. Each stock solution (1.0 mL) was respectively mixed with the autoclaved PDA medium to prepare a set of media containing various concentrations of compounds. Similarly, 0.05% DMSO in culture medium was used as a blank control. The antifungal activity for the concentration of each of the compounds was determined. Each experiment was performed in triplicate. SPSS v17.0 statistics software (SPSS Inc. Released 2008. SPSS Statistics for Windows, Version 17.0. Chicago, IL, USA: SPSS Inc.) was used to analyze the data of the preliminary antifungal activities of three tested compounds at 1 mg/mL. All data were subjected to one-way analysis of variance (ANOVA) using Fisher’s least significant difference (LSD) tests at a *p*-value of <0.05. The average inhibition rate for each test was calculated and transformed to the corresponding probit value. The concentration of the compound was transformed to the corresponding logarithm value ($\log_{10}^{C}$). ${\mathrm{Log}}_{10}^{C}$ values for each compound and its corresponding probit values were used to establish toxicity regression equations by using the least square method. EC_50_ values and their confidence interval at 95% probability were calculated from the toxicity regression equations by using PRISM ver. 5.0 software (GraphPad Software Inc., San Diego, CA, USA).

### 3.6. Assay of in Vivo Antifungal Activity of BAEO by Pot Test

#### 3.6.1. Assay the Control Effect of BAEO against *Gloeosporium orbiculare*

The pot test was fulfilled according to the following steps, which were a traditional and concise method [32]:

**Step 1. Preparation of the tested solution of BAEO.** BAEO was dissolved in 5% DMSO aqueous solution to prepare a set of tested solutions containing 4, 6, 8, 10, 12 mg/mL of BAEO.

**Step 2. Preparation of the spore suspension.** *Gloeosporium orbiculare* was cultured on the PDA medium, then 30 mL of sterilized water was poured into a Petri dish carefully. Finally, appropriate hyphae and spores were scraped onto a Petri dish using an inoculation needle, the spore suspension was obtained after adding a drop of Tween-80. Furthermore, it was ensured that there were 50~60 spores in each view under the microscope.

**Step 3. Cultivation of seedlings.** A certain amount of cucumber seeds (3 rows × 40 lattices) were grown in the seedling dish and cultivated to the late period with two leaves and a heart. The seedlings of approximately the same consistent size were transplanted into the pots to regrow, one seedling in every pot. Twelve pots of cucumber seedlings were prepared in each process.

**Step 4. Protective activity screening.** The inoculation operation was carried out after spraying the BAEO solution for 48 h. Firstly, the prepared spore suspension was evenly spread over both sides of the cucumber leaves with a writing brush—it is worth noting that more suspension should be kept on the obverse surface of the leaf blade. Each experiment was carried out in triplicate. The group only sprayed 5% DMSO on both sides of the cucumber leaves, which was selected as blank controls. Plants were cultivated at 23~25 °C and more than 80% of humidity in a greenhouse, and analyzed after two weeks.

**Step 5. Assay of treatment effectiveness.** BAEO solutions at different concentrations were sprayed evenly onto the infected leaves after they were inoculated for 48 h. Moisturized 24 h later, the tested plants were kept at 23~25 °C and more than 80% of humidity in the greenhouse, and analyzed after two weeks.

**Step 6. Survey of infected condition according to the following classification index:**

Level 0: The area of disease spots is zero.
Level 1: The area of disease spots is below 5% of the total area of infected leaf.
Level 3: The area of disease spots is 6%~10% of the total area of infected leaf.
Level 5: The area of disease spots is 11%~25% of the total area of infected leaf.
Level 7: The area of disease spots is 26%~50% of the total area of infected leaf.
Level 9: The area of disease spots is above 50% of the total area of infected leaf.

**Step 7. Calculation of the control efficacy according to the following equation:**

Disease index (%) = ∑ (*N*_χ_ × *L*_χ_)/(∑*N*_χ_ × *L*_max_) × 100 (2)

where *N*_χ_ refers to the number of infected leaves in different levels, *L*_χ_ is the value of corresponding disease level, and *L*_max_ represents the maximum level of infected leaves.

Control efficacy (%) = (*I*_c_ − *I*_t_)/*I*_c_ × 100 (3)

where *I*_c_ refers to the disease index of the blank control and *I*_t_ represents the disease index of the treated group.

#### 3.6.2. Assay of the Control Effect of BAEO against *Blumeria gramini*s f. sp. *tritici*

In this pot test, the operation process of Steps 1 and 4~7 were totally uniform to those of Section 3.6.1, only Steps 2 and 3 were different and are listed as follows:

**Step 2. Collecting the strain of *Blumeria gramini*s.** Fresh wheat leaves with spores heap of *Blumeria gramini*s were collected in the field, and taken back to maintain wetting for the next step.

**Step 3. Wheat seedlings culture:** One hundred grains of wheat seeds were grown in some disposable paper cups and cultivated to the height of 3~5 cm. The wheat seedlings that were almost in similar height were then transplanted into the pots to regrow, one seedling in every pot. Twelve pots of wheat seedlings were prepared in each process.

## 4. Conclusions

In summary, the present work focused on the extraction, chemical composition, and antifungal activity of essential oil of bitter almond from China, which has been firstly presented by our group. BAEO was readily extracted in a 0.7% yield via hydro-distillation, a succinct and green protocol. Among the 21 types of chemical constituents identified by GC-MS analysis, benzaldehyde (62.52%), benzoic acid (14.80%), and hexadecane (3.97%) ranked the top three. For all tested pathogenic fungi, 1 mg/mL of BAEO showed medium to high activity with inhibition rates that ranged from 44.2 to 100%. The toxicity assay documented that the EC_50_ values of BAEO against *Alternaria brassica* and *Altenraria solani* were only 50.2 and 103.2 mg/mL, respectively, which were higher than those of other fungi. The in vivo antifungal activities against *Gloeosporium orbiculare*, either in considering the protective efficacy or treatment effect, were much better than those of *Blumeria graminis*. The protective efficacy against *Gloeosporium orbiculare* was up to 98.07% at 10 mg/mL and the treatment effectiveness was 93.46% at 12 mg/mL. Based on the results of this work, BAEO could be developed as a botanical and agricultural fungicide in the near future.

## Figures and Tables

**Table 1:** Identification of the chemical composition of bitter almond essential oil (BAEO) by chromatography–mass spectrometry (GC–MS).

Compound	T_R_ (min) ^a^	Formula	CAS Number	Relative Content (%) ^b^	SI (min) ^c^
ethylbenzene	4.495	C_8_H_10_	100-41-4	0.69	93
1,2-xylene	4.560	C_8_H_10_	95-47-6	0.18	89
1,3-xylene	4.720	C_8_H_10_	108-38-3	1.56	95
2-methylundecane	4.865	C_12_H_26_	7045-71-8	0.26	92
tetradecane	5.040	C_14_H_30_	629-59-4	0.61	93
1,4-xylene	5.405	C_8_H_10_	106-42-3	0.90	95
dodecane	5.585	C_12_H_36_	112-40-3	1.50	95
1-ethyl-3-methyl-benzene	6.145	C_9_H_12_	620-14-4	1.61	84
hexadecane	6.345	C_16_H_34_	544-76-3	3.97	94
mesitylene	7.275	C_9_H_12_	108-67-8	1.62	94
1,2,4-trimethylbenzene	7.320	C_9_H_12_	95-63-6	0.23	88
eicosane	10.600	C_20_H_42_	112-95-8	2.26	94
heptadecane	10.705	C_17_H_36_	629-78-7	0.39	94
benzaldehyde	11.955	C_7_H_6_O	100-52-7	62.52	98
dodecyl aldehyde	14.730	C_12_H_24_O	112-54-9	1.18	92
3-(hydroxy-phenyl-methyl)-2,3-dimethyl-octan-4-one	16.025	C_17_H_26_O_2_	911800-56-1	1.23	84
benzyl alcohol	16.865	C_7_H_8_O	100-51-6	1.59	87
benzyl cyanide	17.505	C_8_H_7_N	140-29-4	1.06	92
3-ethyl-2-undecanone	19.995	C_13_H_26_O	328078-05-3	0.50	83
3,5-di-*tert*-butylphenol	21.335	C_14_H_22_O	1138-52-9	1.24	82
benzoic acid	22.835	C_7_H_6_O_2_	65-85-0	14.80	94

^a^ T_R_, retention time listed in the GC–MS spectra; ^b^ Percentage of peak area from the MS signal; ^c^ SI, similarity of the structure between the identified compound and the real one.

**Table 2:** Preliminary antifungal activities of three tested compounds at 1 mg/mL.

Fungi	Average Inhibition Rate ± SD (%) (*n* = 3)		
	BAEO	Benzaldehyde	Kresoxim-Methyl
FOC	68.3 ± 0.9a	62.3 ± 0.5b	67.2 ± 0.9a
VM	50.5 ± 3.0b	51.4 ± 0.0b	81.0 ± 1.6a
PO	95.3 ± 0.6a	54.7 ± 2.3c	82.2 ± 0.2b
FG	87.0 ± 0.3b	81.4 ± 0.4c	93.2 ± 2.3a
AA	80.1 ± 0.2c	86.7 ± 0.1b	93.4 ± 0.1a
AS	100.0 ± 0.0	100.0 ± 0.0	100.0 ± 0.0
PC	49.2 ± 2.7b	39.2 ± 0.9c	85.1 ± 0.4a
GF	91.9 ± 2.0b	100.0 ± 0.0a	100.0 ± 0.0a
FOL	49.5 ± 0.8b	35.5 ± 0.6c	88.8 ± 0.2a
GO	100.0 ± 0.0	100.0 ± 0.0	100.0 ± 0.0
VD	72.5 ± 0.8b	64.2 ± 0.6c	82.3 ± 0.1a
GG	100.0 ± 0.0	100.0 ± 0.0	100.0 ± 0.0
BC	100.0 ± 0.0a	100.0 ± 0.0a	88.8 ± 2.1b
FOV	81.5 ± 0.0	40.7 ± 0.0	63.0 ± 0.0
CL	88.7 ± 0.1b	85.8 ± 0.1c	94.3 ± 0.0a
FOS	46.7 ± 1.7c	74.7 ± 0.8b	81.2 ± 0.5a
CG	44.8 ± 0.6c	55.8 ± 0.5b	100.0 ± 0.0a
FON	56.4 ± 0.4c	83.2 ± 0.2b	100.0 ± 0.0a
AB	100.0 ± 0.0a	28.6 ± 0.0c	83.2 ± 0.8b

For each row, means followed by different lowercase letters were significantly different, which were calculated by one-way analysis of variance (ANOVA) using Fisher’s least significant difference (LSD) test at a *p*-value of <0.05. AA, *Alternaria alternata* (Fr) Keissler; AB, *Alternaria brassicae*; AS, *Alternaria solani*; BC, *Botrytis cinerea*; BG, *Blumeria gramini*s f. sp. *tritici*; CG, *Colletotrichum gloeosporioides* (Penz.) et Sacc.; CL, *Curvularia lunata*; FG, *Fusarium graminearum*; FOC, *Fusarium oxysporum* sp. *cucumebrium* Owen; FOL, *Fusarium oxysporum* f. sp. *lycopersici* Synder et Hansen; FON, *Fusarium oxysporum* f. sp. *niveum*; FOS, *Fusarium* *oxysporum* *(*Schlecht.); FOV, *Fusarium oxysporum* f. sp. *vasinfectum*; GF, *Gloeosporium fructigenum*; GG, *Gaeumannomyces graminis* var. *tritici*; GO, *Gloeosporium orbiculare*; PC, *Phytophthora capsici* Leonian; PO, *Pyricularia oryzae cavgra*; VD, *Verticillium dahliae* Kleb; VM, *Valsa mali* Miyabe et Yamade.

**Table 3:** Toxicity regression equations for concentration effect and median effective concentration (EC_50_) values of BAEO against 19 strains of fungi.

Fungi	Toxicity Regression Equation ^a^	*R*^2^	EC_50_ (μg/mL)	95% CI of LC_50_^b^
FOC	*y* = 1.4963*x* + 0.9404	0.98	511.7	451.4–579.9
VM	*y* = 2.4458*x* − 1.7149	0.93	610.8	531.4–702.1
PO	*y* = 4.0988*x* − 5.7808	0.98	429.3	408.7– 450.9
FG	*y* = 4.3710*x* − 7.1765	0.97	627.9	579.0–680.8
AA	*y* = 2.4905*x* − 1.9416	0.96	642.0	582.6–707.5
AS	*y* = 1.4637*x* + 2.1046	0.96	103.2	73.4–144.9
PC	*y* = 1.5343*x* + 0.7563	0.92	600.5	376.0–959.2
GF	*y* = 2.3698*x* − 0.4715	0.93	225.9	196.7–259.5
FOL	*y* = 1.0912*x* + 2.2974	0.92	295.1	229.1–379.9
GO	*y* = 2.0324*x* + 0.3784	0.94	273.7	230.5–325.0
VD	*y* = 1.9931*x* − 0.0070	0.93	325.2	0.3082–0.5464
GG	*y* = 0.9111*x* + 2.9284	0.98	192.0	104.2–353.7
BC	*y* = 2.0393*x* + 0.5824	0.99	217.0	155.2–303.4
FOV	*y* = 2.2360*x* − 1.0803	0.95	526.7	491.1–564.8
CL	*y* = 1.8933*x* − 0.0960	0.97	509.5	458.2–566.5
FOS	*y* = 2.8836*x* − 2.4579	0.93	423.8	363.8–493.8
CG	*y* = 1.2270*x* + 1.8435	0.98	381.8	353.9–411.8
FON	*y* = 2.2968*x* − 1.2753	0.92	569.3	490.9–660.2
AB	*y* = 0.8082*x* + 3.6361	0.96	50.2	42.4–59.3

^a^ *y*, probability of the inhibition; *x*: log [concentration (mg/mL)]; ^b^ 95% CI, lower and upper values of the confidence interval of the 50% lethal concentration (LC_50_) (mg/mL) at 95% probability.

**Table 4:** Toxicity regression equations for concentration effect and EC_50_ values of kresoxim-methyl against 19 strains of fungi.

Fungi	Toxicity Regression Equation ^a^	*R*^2^	EC_50_ (μg/mL)	95% CI of LC_50_^b^
FOC	*y* = 0.5028*x* + 4.4763	0.95	10.89	8.50–13.97
VM	*y* = 0.5030*x* + 3.9375	0.96	121.20	119.54–120.54
PO	*y* = 0.2362*x* + 4.5941	0.97	51.85	49.31–52.06
FG	*y* = 0.2814*x* + 4.8887	0.98	2.46	1.40–5.59
AA	*y* = 0.2910*x* + 4.7420	0.95	7.737	6.28–9.54
AS	*y* = 1.3929*x* + 4.4564	0.96	2.27	1.95–2.65
PC	*y* = 0.3661*x* + 5.0734	0.95	0.68	0.57–0.85
GF	*y* = 0.7777*x* + 4.4152	0.95	5.87	4.60–6.43
FOL	*y* = 0.4702*x* + 5.0878	0.97	0.71	0.54–0.93
GO	*y* = 0.6174*x* + 4.5918	0.96	4.60	3.96–5.34
VD	*y* = 2.3929*x* +5.4564	0.96	3.78	2.95–4.65
GG	-	-	6.26 ^c^	-
BC	*y* = 0.5430*x* + 3.8613	0.95	132.3	129.81–135.30
FOV	*y* = 0.3288*x* + 4.5625	0.97	21.50	20.34–25.19
CL	*y* = 0.4778*x* + 4.0928	0.96	76.06	74.73–80.36
FOS	*y* = 0.4315*x* + 4.5359	0.98	11.89	10.66–13.25
CG	*y* = 2.3629*x* + 2.1090	0.98	15.45	14.06–16.98
FON	*y* = 0.2123*x* + 4.6062	0.97	71.40	69.81–75.22
AB	*y* = 0.4492*x* + 4.3807	0.98	24.14	21.28–27.38

^a^ *y*, probability of the inhibition; *x*, log [concentration (mg/mL)]; ^b^ 95% CI, lower and upper values of the confidence interval of LC_50_ (mg/mL) at 95% probability; ^c^ Reported in [31].

**Table 5:** The effect of treatment time on the antifungal activity of BAEO against *Fusarium oxysporum* f. sp. *niveum* and *Alternaria* *solani*.

Fungicide	Treatment Time (h)	Inhibition Rate ^a^			
		FON		AS	
		0.5 (mg/mL)	1.0 (mg/mL)	0.5 (mg/mL)	1.0 (mg/mL)
kresoxim-methyl	24	100.00	100.00	50.00	58.33
	48	88.62	91.56	83.03	85.17
	72	68.75	65.33	86.89	90.08
BAEO	24	92.13	100.00	100.00	100.00
	48	53.57	100.00	100.00	100.00
	72	33.33	100.00	100.00	100.00

^a^ Inhibition rate calculated from Equation (1) in Section 3.5.

**Table 6:** Screening the protective effect of BAEO against *Gloeosporium orbiculare*.

Concentration (mg/mL)	Disease Index (%) ^a^	Protective Effect (%) ^b^
4	6.95	88.74
6	5.12	91.33
8	2.96	94.99
10	1.14	98.07
12	11.74	80.13
Control	59.07	-

^a^ Disease index calculated from Equation (2) in Section 3.6.1; ^b^ Protective effect calculated from Equation (3) in Section 3.6.1.

**Table 7:** Assay of the therapeutic effect of BAEO against *Gloeosporium orbiculare*.

Concentration (mg/mL)	Disease Index (%) ^a^	Therapeutic Effect (%) ^b^
4	24.45	58.61
6	25.94	56.09
8	14.36	79.09
10	10.65	81.97
12	3.89	93.41
Control	59.07	-

^a^ Disease index calculated from Equation (2) in Section 3.6.1; ^b^ Therapeutic effect calculated from Equation (3) in Section 3.6.1.

**Table 8:** Screening the protective effect of BAEO against *Blumeria graminis.*

Concentration (mg/mL)	Disease Index (%) ^a^	Protective Effect (%) ^b^
4.0	27.38	50.99
6.0	25.87	53.70
8.0	24.34	56.43
10	23.45	58.03
12	21.56	61.41
Control	55.87	-

^a^ Disease index calculated from Equation (2) in Section 3.6.1; ^b^ Protective effect calculated from Equation (3) in Section 3.6.1.

**Table 9:** Assay of the therapeutic effect of BAEO against *Blumeria graminis*.

Concentration (mg/mL)	Disease Index (%) ^a^	Therapeutic Effect (%) ^b^
4.0	35.37	36.69
6.0	38.89	30.39
8.0	39.85	28.76
10	31.77	43.14
12	27.18	51.35
Control	55.87	-

^a^ Disease index calculated from Equation (2) in Section 3.6.1; ^b^ Therapeutic effect calculated from Equation (3) in Section 3.6.1.

## References

[1] Agrios G.N. (2000). Significance of Plant Diseases.

[2] Lu A., Ma Y., Wang Z., Zhou Z., Wang Q. (2015). Application of “hydrogen-bonding interaction” in drug design. Part 2: Design, synthesis, and structure—Activity relationships of thiophosphoramide derivatives as novel antiviral and antifungal agents. J. Agric. Food Chem..

[3] Bräse S., Encinas A., Keck J., Nising C.F. (2009). Chemistry and biology of mycotoxins and related fungal metabolites. Chem. Rev..

[4] Brent K.J., Hollomon D.W. (1998). Fungicide Resistance: The Assessment of Risk.

[5] Ormancey X., Sisalli S., Coutiere P. (2001). Formulation of essential oils in functional perfumery. Perfum. Cosmet. Actual..

[6] Atiqur R., Sharif M.A., Sun C.K. (2011). Antifungal activity of essential oil and extracts of *Piper chaba* hunter against phytopathogenic fungi. J. Am. Oil Chem. Soc..

[7] Amiri A., Dugas R., Pichot A.L., Bompeix G. (2008). In vitro and in vitro activity of eugenol oil (*Eugenia caryophylata*) against four important postharvest apple pathogens. Int. J. Food Microbiol..

[8] Dikbas N., Kotan R., Dadasoglu F., Sahin F. (2008). Control of *Aspergillus flavus* with essential oil and methanol extract of *Satureja. hortensis*. Int. J. Food Microbiol..

[9] Dubey R.K., Kumar R., Chansouria J.P.N., Dubey N.K. (2008). Evaluation of *Amomum subulatum* Roxb oil as a source of botanical fungitoxicant for the protection of mango fruits from fungal rotting. J. Food Saf..

[10] Tzortzakis N.G. (2009). Impact of cinnamon oil-enrichment on microbial spoilage of fresh produce. Innov.Food Sci. Emerg. Technol..

[11] Tzortzakis N.G., Economakis C.D. (2007). Antifungal activity of lemongrass (*Cympopogon citratus* L.) essential oil against key postharvest pathogens. Innov. Food Sci. Emerg. Technol..

[12] Ma Y., Zhao Z., Li K., Ma X., Guo C., Wei L. (2009). Oil content and composition of almond from different producing area. J. Chin. Cereals Oils Assoc..

[13] Li K., Shi Q., Zhu H., Tang D. (2004). Chemical compositions in bitter almond. J. Northwest For. Univ..

[14] Li K., Shi Q., Zhu H., Tang D. (2003). Study on main nutrient composition of bitter almond. Acta Agric. Boreal. Occident. Sin..

[15] Sun J., Feng X., Zhang X. (1994). Studies of the properties and constituents of almond oil. J. Inn. Mong. Inst. Agric. Anim. Husb..

[16] Ma Y., Zhao Z., Li K., Ma X., Shi Q., Zhu H. (2008). Physicochemical property and fatty acid composition of bitter almond oil. J. Chin. Cereals Oils Assoc..

[17] Li K., Shi Q., Zhu H., Tang D. (2003). Comprehensive exploitation and utilization of bitter almond. J. Northwest For. Univ..

[18] Lim T.K., Lim T.K. (2012). Prunus armeniaca. Edible Medicinal and Non-Medicinal Plants.

[19] Mehdi A., Hamid-Reza K. (2011). Characterization and transesterification of Iranian bitter almond oil for biodiesel production. Appl. Energy.

[20] Korekar G., Stobdan T., Arora R., Yadav A., Singh S.B. (2011). Antioxidant capacity and phenolics content of apricot (*Prunus armeniaca* L.) kernel as a function of genotype. Plant Foods Hum. Nutr..

[21] Ghazavi A., Abtahi H., Karimi M., Mollaghasemi S., Mosayebi G. (2008). Antimicrobial activities of water and methanol extracts of bitter apricot seeds. J. Med. Sci..

[22] Do J.S., Hwang J.K., Seo H.J., Woo W.H., Nam S.Y. (2006). Antiasthmatic activity and selective inhibition of type 2 helper T cell response by aqueous extract of semen *Armeniacae amarum*. Immunopharmacol. Immunotoxicol..

[23] Chang H.K., Shin M.S., Yang H.Y. (2006). Amygdalin induces apoptosis through regulation of Bax and Bcl-2 expressions in human DU145 and LNCaP prostate cancer cells. Biol. Pharm. Bull..

[24] Chang H.K., Yang H.Y., Lee T.H. (2005). *Armeniacae semen* extract suppresses lipopolysaccharide-induced expressions of cyclooxygenase-2 and inducible nitric oxide synthase in mouse BV2 microglial cells. Biol. Pharm. Bull..

[25] Bensky D., Clavey S., Stöger E. (2004). Chinese Herbal Medicine: Materia Medica.

[26] Femenia A., Rossello C., Mulet A., Canellas J. (1995). Chemical composition of bitter and sweet apricot kernels. J. Agric. Food Chem..

[27] Brühne F., Wright E. (2007). Benzaldehyde. Ullmann’s Encyclopedia of Industrial Chemistry.

[28] Lee H., Ahn J., Kwon A., Lee E.S., Kwak J., Min Y. (2014). Chemical composition and antimicrobial activity of the essential oil of apricot seed. Phytother. Res..

[29] Sharma P.C., Gupta A., Kaundal K., Verma A.K. (2013). Antifungal property of essential oil extracted from bitter apricot kernel press cake. Indian Perfum..

[30] Yang R., Gao Z.-F., Zhao J.-Y., Li W.-B., Zhou L., Miao F. (2015). New class of 2-aryl-6-chloro-3,4-dihydroiso-quinolinium salts as potential antifungal agents for plant protection: Synthesis, bioactivity and structure-activity relationships. J. Agric. Food Chem..

[31] Zhang G. (2013). Study on the Chemical Prevention and dsRNA of *Gaeumannomyces graminsis*. Master’s Thesis.

[32] Fang Z.-D. (1998). Methodology for Plant Pathology.

